# Turnover Intention Among Male Nurses in Jiangsu, China: A Structural Equation Modeling Study Based on Social Cognitive Theory

**DOI:** 10.1155/jonm/8865799

**Published:** 2025-05-05

**Authors:** Jianzheng Cai, Yajie Ying, Haifang Wang, Weixia Yu, Yingying Zhang, Sisi Wu

**Affiliations:** ^1^Department of Nursing, The First Affiliated Hospital of Soochow University, Suzhou, China; ^2^School of Nursing, Medical College of Soochow University, Suzhou, China; ^3^Medical Branch, Shanghai Jiao Tong University Press, Shanghai, China

**Keywords:** colleague solidarity, decent work perception, male nurses, social cognitive theory, structural equation model, turnover intention

## Abstract

**Objective:** This study aimed to explore the turnover intention among male nurses and analyze the underlying mechanisms through the lens of the social cognitive theory to provide valuable insights for developing targeted intervention strategies to mitigate the turnover of male nurses.

**Methods:** A cross-sectional survey was conducted. Questionnaires were distributed to male nurses in Jiangsu Province, China, from September to December 2023, with the assistance of the nursing departments. The first part of the questionnaire collected the sociodemographic information. The second part included three instruments, i.e., the Turnover Intention Scale, the Chinese version of the Decent Work Perception Scale, and the Colleague Solidarity Scale for Nurses.

**Results:** Of the 6630 male nurses who were solicited by email, 4227 provided a valid response, and the response rate was 63.76%. Their turnover intention score was 14.27 ± 4.34. According to path analysis, both head nurse support, colleague support, and decent work perception directly reduced turnover intention (*β* = 0.19, 0.47, and −0.65, respectively, *p* < 0.001). However, head nurse support and colleague support indirectly increased turnover intention via decent work perception (*β* = −0.27 and −0.09, respectively, *p* < 0.001).

**Conclusion:** The male nurses in Jiangsu, China, had a high level of turnover intention, and decent work perception and colleague solidarity were key influencing factors. To effectively reduce the turnover intention of male nurses, nursing managers should focus on fostering career development opportunities and cultivating a supportive organizational environment.

## 1. Introduction

The number of male nurses has been steadily increasing over the years, thanks to changing societal perceptions, increased job security in healthcare, and active recruitment efforts targeting men [1]. While the nursing profession is still predominantly female, the percentage of male nurses in registered nurses has reached 40% in Saudi Arabia, 21% in Italy, 11.1% in Australia, 10.7% in the United Kingdom, and 9.1% in the United States [2]. In China, the traditional gender norm is also breaking down, and the ratio of male nurses has increased to 3.4% [3]. Male nurses are often sought after in situations that require heavy lifting or physically demanding tasks, in psychiatric wards, prisons, and substance abuse centers where managing aggressive or violent patients is common, and in military and paramedic roles (e.g., disaster response and flight nursing) [4]. Highly stressful and fast-paced areas such as emergency rooms, intensive care units (ICUs), and trauma centers tend to have a higher number of male nurses, and some male patients may feel more comfortable discussing personal or sensitive health issues (e.g., urological problems, and mental health) with male nurses [5]. However, male nurses tend to have a higher turnover rate than female nurses, due to uncertain career prospects, diminished work value, high workloads, and low social recognition [6]. The estimated turnover rates of male nurse are 9.2% in the United States [7], 37% in Jordan [8], and 38.4% in China [9]. The high turnover rate causes an economic burden for the hospital management, as losing a male nurse costs 0.31–1.3 times his annual salary [10]. The losses negatively impact the organization's stability and productivity, and they also damage nursing quality and patient safety [6]. Consequently, reducing the turnover of male nurses is crucial for mitigating the attrition of the nursing workforce, fostering a healthy nursing profession, and alleviating the financial strain on hospital management.

Turnover intention, which refers to an employee's conscious and deliberate decision to leave their current job to either pursue another opportunity or exit the workforce entirely, is an important predictor of the actual turnover [11]. A significant positive correlation exists between turnover rate and turnover intention, with a correlation coefficient of 0.405 [12]. Hence, exploring the turnover intention among male nurses is crucial for developing strategies to reduce the turnover rate. Existing studies on male nurses' turnover intention have primarily focused on examining the prevalence of the intention across different regions and identifying related influencing factors. Chen et al. [13] conducted a survey among 627 male nurses in China and found that over 80% have a high level of turnover intention. The studies involving 306 male nurses across 16 regions in South Korea and 411 male nurses in Brazil also found high levels of turnover intention [6, 14]. The influencing factors can be categorized into individual factors (e.g., job satisfaction and career growth opportunities), organizational factors (e.g., workplace culture and compensation and benefits), and external factors (e.g., family support and economic conditions) [6, 13, 15]. However, existing studies tend to overlook the interaction between these factors and are often limited to single dimensions, and the mechanisms underlying the turnover intention of male nurses have not been sufficiently explained. To reduce the turnover rate of male nurses, it is critical to explore the mechanisms that drive the turnover intention comprehensively.

**Figure 1:**
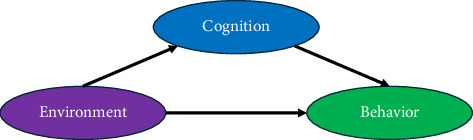
The social cognitive theory model.

Social cognitive theory (SCT) is a psychological framework that explains how individuals learn behaviors, attitudes, and values through observational learning, social interactions, and personal experiences [16]. It has been applied to diverse settings, including education, marketing, and workplace development [17]. It emphasizes the reciprocal relationships between personal factors, environmental influences, and behavior (Figure 1). The key concepts in SCT include reciprocal determinism, observational learning, self-efficacy, reinforcement and punishment, and self-regulation [16]. According to SCT, the external environment shapes the individual's cognition by providing information and context, and cognition ultimately affects behavioral choices as it influences the attitudes and identity related to behavior. In addition, the environment may also directly impact behavioral choices by setting restrictions [16, 18]. According to SCT, some significant factors have been found to affect turnover intention: at the environmental level, leadership style, team-member exchange, and employee trust [19, 20]; at the cognitive level, perceived opportunities for career growth, professional identification, and job satisfaction [21, 22].

Two important concepts that have been examined extensively in nursing management are colleague solidarity and decent work perception (DWP, also “perception of decent work”). These factors influence nurse retention, job performance, patient care quality, and workplace wellbeing [23, 24]. Colleague solidarity refers to the sense of unity, mutual support, and teamwork among nurses within a healthcare setting. It involves head nurse support and colleague support, and it includes trust, collaboration, professional respect, and shared goals that enhance workplace cohesion [23]. With stronger colleague solidarity, nurses tend to have higher job satisfaction, experience less burnout and stress, and are less likely to leave their job [23]. In this way, colleague solidarity improves teamwork and collaboration and enhances patient care. Some key factors that affect colleague solidarity include leadership style, workplace culture, workload and staffing levels, and interpersonal relationships [25]. DWP refers to how nurses evaluate their job conditions in terms of fair wages, job security, work-life balance, professional development, safe and healthy work environment, and workplace respect [26]. Higher levels of DWP also increase job satisfaction, reduce burnout and emotional exhaustion, and improve retention and commitment, all of which contribute to better patient outcomes [24]. These two concepts are intertwined as stronger colleague solidarity can improve DWP and low DWP can weaken colleague solidarity. Nursing management must focus on fostering both aspects to create a sustainable and positive work environment.

In this work, we integrated colleague solidarity and DWP into SCT to formulate a structural equation model (SEM) to analyze the turnover intention of male nurses in Jiangsu, China. The results elucidate the mechanisms underlying the turnover intention of male nurses and offer theoretical guidance in developing strategies to reduce their turnover.

## 2. Methods

### 2.1. Hypotheses

#### 2.1.1. Colleague Solidarity and DWP

Colleague solidarity, which includes both head nurse support and colleague support, captures the level of support, empathy, and cooperation among leaders and peers within the nursing team [23]. Strong colleague solidarity fosters a positive work environment that encourages knowledge sharing and emotional support, which alleviates work-related stress and strengthens professional identity [25]. The DWP encompasses dignity and sense of belonging as it reflects how workers view the work rewards, working conditions, career growth, professional recognition, and the overall work atmosphere [26]. It has been shown that male nurses have a stronger sense of professional belonging and better job satisfaction when their contributions are valued, and their career development is supported [27]. Thus, we assumed that:

  Hypothesis 1a: Head nurse support enhances the level of male nurses' DWP.
  Hypothesis 1b: Colleague support enhances the level of male nurses' DWP.

#### 2.1.2. DWP and Turnover Intention

When the level of DWP is high, the job largely fulfills one's material and spiritual needs. If individuals feel that their work is decent, they tend to invest more in their current job and demonstrate greater loyalty to their organization, and they are inclined to stay [28]. Xue et al. [29] revealed that DWP increases work engagement of nurses and makes them less likely to leave (*β* = −0.062, *p* < 0.001). In addition, DWP also increases job satisfaction and decreases burnout among nurses, thus reducing their turnover intention [24, 30]. Male nurses who feel valued, respected, and have clear career paths are less likely to resign [31]. Taken together, we propose that:

  Hypothesis 2: DWP reduces the turnover intention of male nurses.

#### 2.1.3. Colleague Solidarity and Turnover Intention

Many studies [32, 33] have showed that the support from colleagues and leaders significantly affects turnover intention. Pieretti et al. [34] and Huyghebaert et al. [35] proposed that for healthcare workers, the support from colleagues and the supervisor is negatively associated with turnover intention. Fierce competition among peers and destructive leadership are toxic in the work environment, and they damage one's psychological wellbeing, ultimately increasing turnover intention [36]. Chang et al. [37] found that male nurses with low levels of organizational support are more prone to resignation, particularly after encountering harassment or violence during work. Thus, we hypothesized that:

  Hypothesis 3a: Head nurse support reduces the turnover intention of male nurses.
  Hypothesis 3b: Colleague support reduces the turnover intention of male nurses.

#### 2.1.4. DWP as a Mediator

In SCT, the environment influences behavior by altering one's cognition of behavior, and cognition serves as a mediating factor. Accordingly, we hypothesized that DWP mediates the relationship between turnover intention and head nurse support, as well as the relationship between turnover intention and colleague support. However, there are various other paths between colleague solidarity and turnover intention, and the mediation effect of DWP must be partial. For example, higher colleague solidarity has been correlated with stronger organizational commitment, which reflects a healthy relationship between the organization and its members and is directly related to turnover intention [38]. Similarly, organizational support has been identified as a determinant of burnout [39], and a direct relationship exists between burnout and turnover intention [40]. Therefore, based on these arguments, we propose the following two hypotheses.

  Hypothesis 4a: DWP partially mediates the relationship between head nurse support and turnover intention.
  Hypothesis 4b: DWP partially mediates the relationship between colleague support and turnover intention.

**Figure 2:**
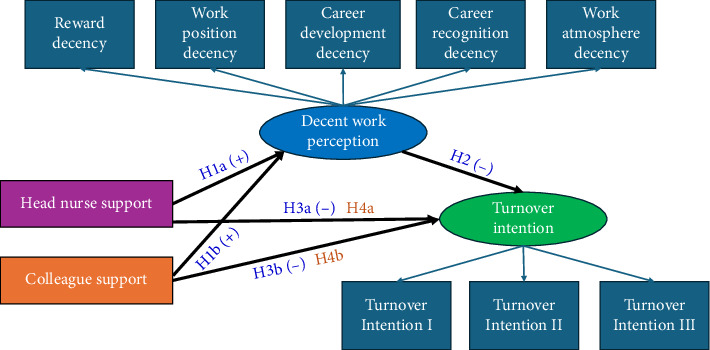
Hypothesized theoretical model. H1a, H1b, H2, H3a, and H3b are direct effects. H4a and H4b denote an indirect effect.

Figure 2 summarizes the SCT framework with the outlined hypotheses.

### 2.2. Settings

From September to December 2023, a cross-sectional survey was conducted among clinical male nurses working in medical institutions across Jiangsu, China. Hospitals were included if they employed registered male nurses and participated voluntarily. With a reference letter from the Jiangsu Nursing Association, we contacted the leadership of nursing departments (mainly directors or deputy directors) across 2132 hospitals in Jiangsu and explained the aim and significance of this study. With the approval and assistance of the nursing departments, our study was publicized in their hospitals, and emails were sent to the registered male nurses working at the respective institutions, who were (1) currently employed as a nurse with a valid qualification certificate and (2) engaging actively in clinical nursing work. Male nurses who were visiting for advanced studies or working as an intern were excluded. Two emails were sent, with the second one 10 days after the first one as a reminder.

The sample size was determined based on the rule-of-thumb, which recommends a ratio of 5–10 samples per variable for SEM [41]. This study examined a total of 24 variables, with 14 related to demographics and 3, 5, and 2 to three separate scales. Assuming an attrition rate of 20%, the minimum sample size of this work was 5 × 24 × (1 + 20%) = 144.

### 2.3. Data Collection

Qualifying male nurses were solicited to participate anonymously and voluntarily. They were given a link to a questionnaire, which consisted of two parts, hosted on Wenjuanxing (https://www.wjx.cn/), a reputable online survey platform in China. A translated version of the questionnaire is given in Supporting Information 1. The first part of the questionnaire explained the purpose and significance of the study, emphasized the right to withdraw at any time, and asked the participants to provide their informed consent. Only after all confirmations and agreements were cleared, one could proceed to the next part. The second part inquired about their sociodemographic information (with 14 variables) and then presented three instruments (described below). The demographic section was developed after a thorough literature review and aligned with the objectives of this study.

The questionnaire could not be submitted until all entries were filled. Questionnaires submitted within 4 weeks of the second email were considered, while those completed in under 2 min were excluded [42]. The STROBE reporting guidelines [43] were followed (Supporting Information 2: Supporting Table 1), and the collected data were used exclusively for this study's analysis.

### 2.4. Instruments

#### 2.4.1. Turnover Intention Scale (TIS)

The TIS, which utilizes self-assessment to measure the turnover intention of an employee, was developed by Michaels and Spector and translated and revised by Li et al. [44]. It consists of three subscales, i.e., TIS I, TIS II, and TIS III, which assess the likelihood of quitting the current job, the motivation to seek other employment, and the actual pursuit of outside job opportunities, respectively. Items are rated on a four-point Likert scale, ranging from 1 (*never*) to 4 (*always*). The total score is between 6 and 24, and a higher score reflects a stronger turnover intention. In Li et al.'s study, the Cronbach's α of TIS is 0.745 [44].

#### 2.4.2. Decent Work Perception Scale, Chinese Version (DWPS)

This scale was developed by Mao et al. [45]. It consists of 16 items and measures how the workers perceive the decency of their workplace from five dimensions, i.e., reward decency, work position decency, career development decency, career recognition decency, and work atmosphere decency. The responses are recorded on a five-point Likert scale, with scores ranging from 1 (*strongly disagree*) to 5 (*strongly agree*), and Items 5, 6, 7, and 8 are reverse scored. The total score ranges from 16 to 80, and higher scores indicate greater DWP. Mao et al. verified the reliability and validity of the revised questionnaire, which gives a Cronbach's α of 0.745 and a retest reliability of 0.775 [45].

#### 2.4.3. Colleague Solidarity Scale for Nurses (CSSN)

The CSSN was originally developed by Greene and later translated into Chinese by Gao et al. [46]. It consists of two subscales. Subscale A has nine items and evaluates the support provided by head nurses. Subscale B has 21 items and assesses the level of support among nursing colleagues. A five-point Likert scale with reverse scoring is used, and the scores range from 1 (*always*) to 5 (*never*). That is, the total score ranges from 30 to 150, and higher scores indicate lower levels of colleague solidarity. The total Cronbach's α is 0.892, 0.78, and 0.89 for the original Subscale A, Subscale B, and CSSN, respectively [46].

### 2.5. Ethical Consideration

This study complied with the Declaration of Helsinki and was approved by the Medical Ethics Committee of the First Affiliated Hospital of Soochow University (Approval No. 2022535). Informed consent was obtained from all participants.

### 2.6. Data Analysis

Data were expressed as mean ± standard deviation or count (ratio) and analyzed using SPSS 26.0. The Pearson correlation analysis was employed to examine the relationships between DWP, colleague solidarity, and turnover intention. AMOS 26.0 was utilized for path analysis to evaluate the fit of the hypothesized model. The maximum likelihood estimation was used in path analysis to validate the associations and predictions of the model. To assess the significance of indirect effects, bootstrapping was conducted with 5000 samples, and the bias-corrected confidence interval (CI) was 95%. The following indices were used to assess the goodness of fit of the model: *p* value (*p* < 0.05), normalized chi-square (*χ*^2^/df < 5), root mean square error of approximation (RMSEA ≤ 0.08), goodness of fit index (GFI ≥ 0.90), adjusted goodness of fit index (AGFI ≥ 0.90), normed fit index (NFI ≥ 0.90), incremental fit index (IFI > 0.90), Tucker–Lewis index (TLI ≥ 0.95), comparative fit index (CFI ≥ 0.95), and standardized root mean square residual (SRMR ≤ 0.05).

## 3. Results

### 3.1. Questionnaire Response

Of the 2132 hospitals we contacted, 762 met the eligibility criteria. They included 206 tertiary, 483 secondary, 27 primary, and 46 specialized hospitals, and they were distributed across all 13 prefecture-level administrative divisions in the Jiangsu Province, i.e., Nanjing (138 hospitals), Suzhou (151 hospitals), Wuxi (72 hospitals), Changzhou (31 hospitals), Yangzhou (52 hospitals), Nantong (82 hospitals), Yancheng (35 hospitals), Lianyungang (24 hospitals), Huai'an (34 hospitals), Suqian (29 hospitals), Xuzhou (47 hospitals), Taizhou (31 hospitals), and Zhenjiang (36 hospitals). There were 6630 registered male nurses working at the eligible hospitals, all of whom received our request to participate. We received valid responses from 4227 male nurses, and the response rate was 63.76%.

### 3.2. Demographics and Assessment Results

Table 1 shows that of the 4227 male nurses, the age was 27.34 ± 4.85 years, and the work experience was 5.26 ± 4.30 years. Most of them had a bachelor's degree (61.65%), did not marry (61.51%), were themselves the only child (54.98%), and did not have their own children (71.75%). In addition, 82.35% had a primary title, 78.92% were contract workers, and 60.70% worked at tertiary hospitals. They showed a high level of turnover intention, as the total scores for turnover intention, DWP, head nurse support, and colleague support were 14.27 ± 4.34, 49.29 ± 6.53, 33.95 ± 7.89, and 79.83 ± 16.04, respectively (Table 2).

### 3.3. Correlation Analysis

Table 3 shows the Pearson correlation analysis of turnover intention, DWP, and colleague solidarity among male nurses. The turnover intention was correlated positively with head nurse support (*r* = 0.26, *p* < 0.01) and colleague support (*r* = 0.31, *p* < 0.01) but negatively with DWP (*r* = −0.33, *p* < 0.01). Furthermore, a positive correlation existed between head nurse support and DWP (*r* = 0.39, *p* < 0.01) and between colleague support and DWP (*r* = 0.36, *p* < 0.01).

### 3.4. Structural Equation Modeling

#### 3.4.1. Model Fitting

An initial SEM was constructed to test the four hypotheses (Supporting Information 2: Supporting Figure 1). The maximum likelihood method was employed to fit the model to the data (Table 4). The goodness of fit measures were as follows: *χ*^2^/df = 253.45 (poor), RMSEA = 0.23 (poor), GFI = 0.65 (poor), AGFI = 0.61 (poor), NFI = 0.65 (poor), IFI = 0.65 (poor), TLI = 0.51(poor), CFI = 0.65 (poor), and SRMR = 0.18 (poor). Further model tuning was deemed necessary to improve the model fitness.

#### 3.4.2. Model Modification

**Figure 3:**
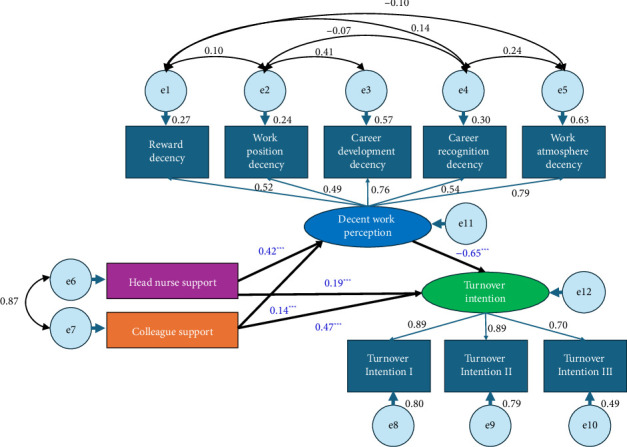
Refined structural equation model for the turnover intention of male nurses. ^∗∗∗^*p* < 0.001.

To improve the model fitting, the initial model was refined by applying modification indices (MIs) to incorporate the relationships suggested by AMOS [47]. Because SEM is a theoretically driven technique, we only added theoretically meaningful modifications.  Figure 3 illustrates the final model and the specific modification, with the fitness indicators being *χ*^2^/df = 50.37 (poor), RMSEA = 0.11 (poor), GFI = 0.95 (good), AGFI = 0.88 (acceptable), NFI = 0.95 (good), IFI = 0.95 (good), TLI = 0.90 (good), CFI = 0.95 (good), and SRMR = 0.09 (poor). In SEM, when the sample size is large, small deviations between the model and the data occur easily, and it is hard for *χ*^2^/df to reach an “ideal” value (typically less than 5) because the index is overly sensitive to those deviations. That is, with larger sample sizes, *χ*^2^/df becomes less useful for assessing model fitness [48]. After considering the model fit indices comprehensively, the final model was deemed acceptable and worthy of discussion.

#### 3.4.3. Path Analysis

With the refined SEM, path coefficient estimation and significance testing were carried out to verify the relationships between variables.  Table 5 shows that head nurse support and colleague support had a direct impact on DWP (*β* = 0.42 and 0.14, respectively, *p* < 0.001). However, because CSSN adopted reverse scoring, head nurse support and colleague support were in fact negatively correlated with DWP, which contradicted H1a and H1b. Moreover, the turnover intention was influenced directly by DWP (*β* = −0.65, *p* < 0.001), head nurse support (*β* = 0.19, *p* < 0.001), and colleague support (*β* = 0.47, *p* < 0.001), which verified H2, H3a, and H3b.

#### 3.4.4. Effect Analysis

The bootstrap method was employed to ascertain the mediating effect of DWP on the relationship between head nurse support and turnover intention, as well as colleague support and turnover intention (Table 6). The lower and upper bounds of the mediating effect between head nurse support and turnover intention were −0.32 and −0.22, respectively, while those between colleague support and turnover intention were −0.14 and −0.05, respectively. Since the 95% CI did not include 0, the indirect effect could be established, and both H4a and H4b were confirmed. Both head nurse support (indirect effect = −0.27, accounting for 143.16% of the direct effect) and colleague support (indirect effect = −0.09, accounting for 19.53% of the direct effect) indirectly influenced turnover intention through DWP.

## 4. Discussion

### 4.1. A Snapshot of Turnover Intention Among Male Nurses in Jiangsu, China

The 4227 male nurses who participated in this study had a high level of turnover intention. Their TIS score (14.27 ± 4.34) was lower than that of the 627 male nurses across 331 hospitals in China in Chen's survey [13] but higher than the scores reported for the United States and Belgium [7, 8]. The following factors may have contributed to the high turnover intention among Chinese male nurses.

Firstly, developed countries have a long history of male nurses and have established robust talent cultivation systems. Male nurses in these countries generally have higher salaries, better benefits, and more opportunities for career advancement [49]. In contrast, China is an Asian country influenced by Confucian values, and the societal expectation is that men should pursue challenging professions with high income to fulfill their role as the provider of their family [50]. The social status and professional image of nursing are often in conflict with traditional notions of masculinity, which reduce the recognition from the family and the society [51] and thus increase the turnover intention.

In addition, male nurses are predominantly employed in high-stress environments such as operating rooms, emergency departments, and ICUs [52] where they routinely serve patients with critical and life-threatening conditions. In this study, many male nurses worked in these settings (operating room, 17.29%; emergency department, 22.69%; ICU, 24.46%), and they worked many night shifts each month (1–4, 30.59%; 5–9, 34.33%; ≥ 10, 19.97%). The demanding nature of their work likely contributes to the turnover intention [53, 54].

Hence, to mitigate turnover intention of male nurses, the hospital management should focus on revising training and development systems, improving work environments, and ensuring equitable work assignments. Meanwhile, establishing role models of male nurses within the profession may reduce turnover intention by strengthening professional identity and motivation.

### 4.2. Mechanisms Underlying Turnover Intention

#### 4.2.1. Decent Work Perception Had a Direct Negative Influence on Turnover Intention

Among the surveyed male nurses, the level of DWP had a significant direct negative effect on the turnover intention, with a path coefficient of −0.74. The results agreed well with the findings of Chada et al. [55] and indicated that higher levels of DWP were associated with lower turnover intention. The observation can be understood in the following context. When male nurses consider their job as a decent work that fulfills both their material and psychological needs, they are more likely to invest time and energy into their roles [56]. This investment enhances their job satisfaction and the sense of self-worth, thereby reducing their inclination to leave [29]. Conversely, when male nurses feel that their work lacks decency, they are more likely to experience a “pay-reward imbalance.” Consequently, they experience poorer sense of belonging, diminished professional identity, and detachment from their jobs [57], all of which significantly increase their turnover intention [30].

A few measures are expected to elevate the DWP of male nurses and reduce their turnover intention effectively. First, positive media coverage is crucial to enhancing the societal awareness and recognition of male nurses. Within the hospital, nursing management should foster an environment of mutual respect, thereby improving the professional recognition of male nurses. Implementing a fair and motivating salary system is essential to incentivize and fully engage male nurses. Developing clear career advancement plans, including opportunities for further education and professional growth, will also help enhance DWP.

#### 4.2.2. Colleague Solidarity Had Direct and Indirect Effects on Turnover Intention

Colleague solidarity had both direct and indirect effects on the turnover intention of male nurses. The direct effect of head nurse support and colleague support on turnover intention was 0.19 and 0.47, respectively, suggesting that stronger colleague solidarity (i.e., lower score, because CSSN adopts reverse scoring) reduced turnover intention. The results were consistent with the findings of Hämmig [58] and Chen et al. [59]. When male nurses receive strong support from head nurses and colleagues, they become more confident in tackling challenges, are more inclined to seek help, adopt positive coping strategies, and develop resilience to stress [60], all of which contribute to lower turnover intention. Interventions that focus on department-wide support and strong organizational backing effectively reduce the turnover intention of nurses [61]. The present findings indicate that nurse managers should communicate timely with male nurses to understand and address their needs in career development and provide them with personalized career support. Within departments, to encourage collaborative efforts that lead to shared achievements, nurse managers also need to foster a culture of mutual support and cooperation among colleagues. These forms of support not only strengthen the self-efficacy of male nurses but also reduce their inclination to leave the profession.

However, head nurse support and colleague support aggravated turnover intention when the mediation of DWP was considered, and the coefficient of this suppressor effect was −0.27 and −0.09, respectively. Regarding head nurse support, Yang et al. [62] found that when the nursing administrators' support fails to align with the substantive needs of male nurses, particularly when it does not manifest as tangible institutional assurance for career progression, equitable remuneration systems, or professional development, the support may be perceived as perfunctory and damage the career development expectation of male nurses. Moreover, ostensibly supportive interventions are always accompanied by higher job demands or hidden pressure. Heightened scrutiny of daily tasks or the assignment of additional managerial or technical responsibilities can be perceived as excessive interference, thereby exacerbating psychological attrition, further reducing DWP and exacerbating turnover intention [63].

For colleague support, nursing is a female-dominated profession, and male nurses are a minority. This minority status can lead to different experiences in career progression and social recognition. Gauci et al. [64] argued in an integrative review that while male nurses may receive promotions and opportunities due to their gender, they also face challenges such as discrimination stemming from gender stereotypes (careless, impatient, and less compassionate), isolation, and disproportionate physical labor in their working environment, which lead to increased job stress and burnout [65]. Baker et al. [66] found that male nurses in Australia are conscious that patients regard nursing as a female profession and see them as potential sexual threats. Hence, in the context of DWP, when male nurses compare themselves to their female peers, they may sense a comparative disadvantage incentivizing them to leave. Therefore, nurse managers should strive to offer gender-equal development opportunities and foster an atmosphere of equality. In addition, career planning support should be provided within appropriate limits to avoid the detrimental effects of excessive intervention.

### 4.3. Limitations

This study was conducted in Jiangsu, which is an economically developed province of China. The situation in other areas with different economic conditions will likely vary. Therefore, cross-regional and multicenter studies should be conducted in the future. Secondly, this study relied on self-report questionnaires and was thus susceptible to various typical limitations, including subjectivity, bias, question phrasing, and lack of verification.

## 5. Conclusions

Through SCT, this study revealed that the male nurses in Jiangsu, China, had high turnover intention. Both head nurse support, colleague support, and DWP directly reduced turnover intention, but head nurse support and colleague support indirectly increased turnover intention via DWP. To decrease the turnover rate of male nurses, nursing managers should foster a collaborative team environment, enhance support systems, offer personalized career development plans, and improve the DWP among male nurses.

## Figures and Tables

**Table 1:** Overview of study subjects (*n* = 4227).

Item	*n* (%)
*Age (years)*	
< 30	3010 (71.21)
30–35	899 (21.27)
> 35	318 (7.52)

*Education level*	
Doctor	6 (0.14)
Master	60 (1.42)
Bachelor	2606 (61.65)
College diploma	1532 (36.24)
Technical secondary school and below	23 (0.54)

*Marital status*	
Single	2600 (61.51)
Married	1600 (37.85)
Divorced and others	27 (0.64)

*Only child*	
Yes	2324 (54.98)
No	1903 (45.02)

*Family size*	
No children	3033 (71.75)
One child	915 (21.65)
Two or more children	279 (6.60)

*Work experience (years)*	
< 5	2313 (54.72)
5–10	1334 (31.56)
> 10	580 (13.72)

*Annual income (RMB)*	
≤ 50,000	839 (19.85)
60,000–100,000	1823 (43.13)
110,000–150,000	1153 (27.28)
160,000–200,000	337 (7.97)
210,000–250,000	63 (1.49)
> 250,000	12 (0.28)

*Job* *title*^†^	
Primary title	3481 (82.35)
Middle title	682 (16.13)
Advanced title	64 (1.51)

*Employment type*	
Permanent employees	386 (9.13)
Contract worker	3336 (78.92)
Dispatched labor	505 (11.95)

*Hospital type*	
Tertiary hospital	3488 (60.70)
Secondary hospital	631 (14.93)
Primary hospital	32 (0.76)
Specialized hospital	76 (1.80)

*Department*	
Internal medicine	330 (7.81)
Surgery	327 (7.74)
Emergency	959 (22.69)
Outpatient	41 (0.97)
ICU	1034 (24.46)
Operating room	731 (17.29)
Anesthesiology	62 (1.47)
Pediatrics	26 (0.62)
Hemodialysis	106 (2.51)
Others	611 (14.45)

*Clinical teacher*	
Yes	598 (14.15)
No	3629 (85.85)

*Clinical nurse specialist*	
No	3806 (90.04)
Municipal level	195 (4.61)
Provincial level	159 (3.76)
National level	41 (0.97)
Others	26 (0.62)

*Night shifts (times per month)*	
0	639 (15.12)
1–4	1293 (30.59)
5–9	1451 (34.33)
≥ 10	844 (19.97)

^†^Primary title, nurse and senior nurse; middle title, supervising nurse; advanced title, associate chief nurse and chief nurse.

**Table 2:** Total scores of each scale and scores of each dimension.

Instrument^†^	Dimension	Items	Result^§^	Cronbach's α^¶^
TIS	Overall score	6	14.27 ± 4.34	0.898
	Turnover Intention Scale I	2	4.69 ± 1.71	
	Turnover Intention Scale II	2	4.64 ± 1.59	
	Turnover Intention Scale III	2	4.49 ± 1.53	

DWPS	Overall score	16	49.29 ± 6.53	0.935
	Reward decency	4	10.70 ± 2.79	
	Work position decency	3	9.23 ± 3.06	
	Career development decency	3	10.01 ± 2.46	
	Career recognition decency	3	8.93 ± 2.77	
	Work atmosphere decency	3	11.15 ± 2.56	

CSSN	Overall score	30	113.77 ± 22.90	0.989
	Head nurse support	9	33.95 ± 7.89	0.983
	Colleague support	21	79.83 ± 16.04	0.987

^†^TIS, Turnover Intention Scale, total score is 6–24 and higher scores reflect stronger turnover intention; DWPS, Decent Work Perception Scale, total score is 16–80 and higher scores reflect stronger decent work perception; CSSN, Colleague Solidarity Scale for nurses, total score is 30–150 and higher scores reflect lower levels of colleague solidarity.

^§^Expressed as mean ± standard deviation.

^¶^Calculated based on the results of this study.

**Table 3:** Correlations between variables (*n* = 4227).

Item^†^		TIS				DWPS						CSSN		
		1	2	3	4	5	6	7	8	9	10	11	12	13
TIS	1	1												
	2	0.799	1											
	3	0.624	0.628	1										
	4	0.914	0.910	0.841	1									

DWPS	5	−0.363	−0.310	−0.221	−0.337	1								
	6	−0.363	−0.324	−0.249	−0.353	0.349	1							
	7	−0.332	−0.279	−0.159	−0.291	0.422	0.605	1						
	8	−0.234	−0.159	−0.058	−0.172	0.384	0.205	0.414	1					
	9	−0.126	−0.059	0.040	−0.057	0.361	0.356	0.591	0.556	1				
	10	−0.386	−0.308	−0.176	−0.329	0.686	0.690	0.818	0.696	0.782	1			

CSSN	11	0.171	0.228	0.287	0.256	0.116	0.147	0.347	0.263	0.546	0.386	1		
	12	0.224	0.283	0.327	0.311	0.101	0.123	0.316	0.251	0.518	0.356	0.868	1	
	13	0.213	0.273	0.324	0.302	0.109	0.134	0.336	0.262	0.543	0.377	0.938	0.986	1

*Note:* All entries satisfy *p* < 0.01.

^†^1, Turnover Intention Scale (TIS) I; 2, TIS II; 3, TIS III; 4, overall TIS score; 5, reward decency; 6, work position decency; 7, career development decency; 8, career recognition decency; 9, work atmosphere decency; 10, overall Decent Work Perception Scale (DWPS) score; 11, head nurse support; 12, colleague support; 13, overall Colleague Solidarity Scale for Nurses (CSSN) score.

**Table 4:** Goodness of fit of the models.

Index^†^	*χ* ^2^/df	RMSEA	GFI	AGFI	NFI	IFI	TLI	CFI	SRMR
Benchmark	< 5	< 0.08	> 0.90	> 0.90	> 0.90	> 0.90	> 0.90	> 0.90	< 0.05

Initial model	253.447	0.224	0.648	0.610	0.647	0.648	0.505	0.648	0.179
Refined model	50.373	0.108	0.946	0.881	0.945	0.946	0.903	0.946	0.091

^†^ *χ* ^2^/df, normalized chi-square; RMSEA, root mean square error of approximation; GFI, goodness of fit index; AGFI, adjusted goodness of fit index; NFI, normed fit index; IFI, incremental fit index; TLI, Tucker–Lewis index; CFI, comparative fit index; SRMR, standardized root mean square residual.

**Table 5:** Path analysis.

Path	Effect (unstandardized *β*)	SE^†^	CR^†^	*p*	Effect (standardized *β*)
Head nurse support ⟶ decent work perception	0.063	0.005	12.779	< 0.001	0.417
Colleague support ⟶ decent work perception	0.010	0.002	4.523	< 0.001	0.140
Decent work perception ⟶ turnover intention	−0.686	0.032	−21.714	< 0.001	−0.650
Head nurse support ⟶ turnover intention	0.030	0.005	6.350	< 0.001	0.190
Colleague support ⟶ turnover intention	0.035	0.002	16.216	< 0.001	0.466

^†^SE, standard error; CR, critical ratio.

**Table 6:** Intermediary effect between colleague solidarity and turnover intention.

Path/effect	Effect	95% confidence interval
Head nurse support ⟶ decent work perception	0.417	[0.350, 0.482]
Colleague support ⟶ decent work perception	0.140	[0.072, 0.208]
Decent work perception ⟶ turnover intention	−0.650	[−0.696, −0.605]

*Head nurse support ⟶ turnover intention*		
Direct effect	0.190	[0.124, 0.253]
Indirect effect (via decent work perception)^§^	−0.272	[−0.321, −0.223]
Ratio of the indirect effect to the direct effect	143.16%	—
Total effect	−0.082	[−0.154, −0.011]

*Colleague support ⟶ turnover intention*		
Direct effect	0.466	[0.403, 0.530]
Indirect effect (via decent work perception)^¶^	−0.091	[−0.136, −0.046]
Ratio of the indirect effect to the direct effect	19.53%	—
Total effect	0.314	[0.304, 0.445]

^§^Head nurse support ⟶ decent work perception ⟶ turnover intention.

^¶^Colleague support ⟶ decent work perception ⟶ turnover intention.

## Data Availability

Detailed data that support the findings of this study are not publicly available due to privacy concerns and ethical restrictions but are available from the authors upon request.

## Supporting Information

Supporting Information 1 Supporting Information 1: Informed consent and the translated questionnaire.

Supporting Information 2 Supporting Information 2: Figure 1. Initial structural equation model for the turnover intention of male nurses. Table 1. STROBE Statement: Checklist of items that should be included in reports of cross-sectional studies.

## References

[1] Martsolf G. R., Gigli K., Case B., Dill J., Dierkes A. (2023). Describing the Male Registered Nursing Workforce Toward Increasing Male Representation in Professional Nursing. *Nursing Outlook*.

[2] Younas A., Ali N., Sundus A., Sommer J. (2022). Approaches of Male Nurses for Degendering Nursing and Becoming Visible: A Metasynthesis. *Journal of Clinical Nursing*.

[3] National Health Commission of China (2022). *China Health Yearly Statistics 2022*.

[4] Trudeau R. (1996). Male Registered Nurses. *Health Reports*.

[5] Zhang X., Zhang B., Meng P. (2019). Work Experience of Male Nurses in Andrology: A Qualitative Study. *National journal of andrology*.

[6] Kim S., Moon S. (2021). Factors Influencing Turnover Intention Among Male Nurses in Korea. *International Journal of Environmental Research and Public Health*.

[7] Bruyneel A., Bouckaert N., Maertens de Noordhout C. (2023). Association of Burnout and Intention-To-Leave the Profession With Work Environment: A Nationwide Cross-Sectional Study Among Belgian Intensive Care Nurses After Two Years of Pandemic. *International Journal of Nursing Studies*.

[8] Hayajneh Y. A., AbuAlRub R. F., Athamneh A. Z., Almakhzoomy I. K. (2009). Turnover Rate Among Registered Nurses in Jordanian Hospitals: An Exploratory Study. *International Journal of Nursing Practice*.

[9] Young M. D., Plotnikoff R. C., Collins C. E., Callister R., Morgan P. J. (2014). Social Cognitive Theory and Physical Activity: A Systematic Review and Meta-Analysis. *Obesity Reviews*.

[10] Li Y., Jones C. B. (2013). A Literature Review of Nursing Turnover Costs. *Journal of Nursing Management*.

[11] Elfios E., Asale I., Merkine M. (2024). Turnover Intention and Its Associated Factors Among Nurses in Ethiopia: A Systematic Review and Meta-Analysis. *BMC Health Services Research*.

[12] Lee Y., Dai Y., Chang M. (2017). Quality of Work Life, Nurses’ Intention to Leave the Profession, and Nurses Leaving the Profession: A One‐Year Prospective Survey. *Journal of Nursing Scholarship*.

[13] Chen J., Dai Y., Qin Y. (2024). Factors Influencing Turnover Intention Among Male Nurses in China: A Large-Scale Descriptive Correlational Study. *International Nursing Review*.

[14] Mao A., Wang J., Zhang Y., Cheong P. L., Van I. K., Tam H. L. (2020). Factors Influencing Recruitment and Retention of Male Nurses in Macau and Mainland China: A Collaborative, Qualitative Study. *BMC Nursing*.

[15] de Oliveira D. R., Griep R. H., Portela L. F., Rotenberg L. (2017). Intention to Leave Profession, Psychosocial Environment and Self-Rated Health Among Registered Nurses from Large Hospitals in Brazil: A Cross-Sectional Study. *BMC Health Services Research*.

[16] Bandura A. (2001). Social Cognitive Theory: An Agentic Perspective. *Annual Review of Psychology*.

[17] de la Fuente J., Kauffman D. F., Boruchovitch E. (2023). Editorial: Past, Present and Future Contributions From the Social Cognitive Theory (Albert Bandura). *Frontiers in Psychology*.

[18] Bussey K., Bandura A. (1999). Social Cognitive Theory of Gender Development and Differentiation. *Psychological Review*.

[19] Iqbal J., Asghar A., Asghar M. Z. (2022). Effect of Despotic Leadership on Employee Turnover Intention: Mediating Toxic Workplace Environment and Cognitive Distraction in Academic Institutions. *Behavioral Sciences*.

[20] Pham T. T. L., Teng C., Friesner D. (2019). The Impact of Mentor-Mentee Rapport on Nurses’ Professional Turnover Intention: Perspectives of Social Capital Theory and Social Cognitive Career Theory. *Journal of Clinical Nursing*.

[21] Deng H., Guan Y., Zhou X. (2024). The “Double-Edged Sword” Effects of Career Support Mentoring on Newcomer Turnover: How and When it Helps or Hurts. *Journal of Applied Psychology*.

[22] Zhang X., Deng H., Xia Y., Lan Y. (2021). Employability Paradox: The Effect of Development Idiosyncratic Deals on Recipient Employees’ Turnover Intention. *Frontiers in Psychology*.

[23] Wang J., Liu S., Qu X. (2023). Nurses’ Colleague Solidarity and Job Performance: Mediating Effect of Positive Emotion and Turnover Intention. *Safety and Health at Work*.

[24] Sönmez B., Yıldız Keskin A., İspir Demir Ö., Emiralioğlu R., Güngör S. (2023). Decent Work in Nursing: Relationship between Nursing Work Environment, Job Satisfaction, and Physical and Mental Health. *International Nursing Review*.

[25] Göktepe N., Yalçın B., Türkmen E., Dirican Ü., Aydın M. (2020). The Relationship Between Nurses’ Work-Related Variables, Colleague Solidarity and Job Motivation. *Journal of Nursing Management*.

[26] Seubert C., Hopfgartner L., Glaser J. (2021). Living Wages, Decent Work, and Need Satisfaction: An Integrated Perspective. *European Journal of Work & Organizational Psychology*.

[27] Lee S. S., Lee H. J., Jung H. (2021). The Effect of Hospital Organizational Culture on Job Satisfaction of Male Nurses. *Korean Journal of Occupational Health*.

[28] Feng Y., Li S., Ma W. (2025). The Relationship Between Compassion Fatigue, Engagement and Decent Work Among Nurses: A Cross-Sectional Study. *BMC Nursing*.

[29] Xue B., Feng Y., Zhao Y. (2024). Decent Work, Work Engagement, and Turnover Intention Among Registered Nurses: A Cross-Sectional Study. *BMC Nursing*.

[30] Xue B., Feng Y., Hu Z. (2024). Assessing the Mediation Pathways: How Decent Work Affects Turnover Intention Through Job Satisfaction and Burnout in Nursing. *International Nursing Review*.

[31] Valizadeh L., Zamanzadeh V., Fooladi M. M., Azadi A., Negarandeh R., Monadi M. (2014). The Image of Nursing, as Perceived by Iranian Male Nurses. *Nursing and Health Sciences*.

[32] Liu Y., Duan Y., Guo M. (2023). Turnover Intention and Its Associated Factors Among Nurses: A Multi-Center Cross-Sectional Study. *Frontiers in Public Health*.

[33] Janusik L. (2023). Listening Training in Organizations. *Current Opinion in Psychology*.

[34] Pieretti A., Bastiani L., Bellandi T., Molinaro S., Zoppi P., Rasero L. (2022). Second Victim Experience and Support Tool: An Assessment of Psychometric Properties of Italian Version. *Journal of Patient Safety*.

[35] Huyghebaert T., Gillet N., Audusseau O., Fouquereau E. (2019). Perceived Career Opportunities, Commitment to the Supervisor, Social Isolation: Their Effects on Nurses’ Well-Being and Turnover. *Journal of Nursing Management*.

[36] Trépanier S., Peterson C., Fernet C., Austin S. (2024). How Tyrannical Leadership Relates to Workplace Bullying and Turnover Intention over Time: The Role of Coworker Support. *Scandinavian Journal of Psychology*.

[37] Chang H., Jeong S. (2021). Male Nurses’ Experiences of Workplace Gender Discrimination and Sexual Harassment in South Korea: A Qualitative Study. *Asian Nursing Research*.

[38] Yılmaz Y., Üngüren E., Tekin Ö. A., Kaçmaz Y. Y. (2022). Living with Infection Risk and Job Insecurity during COVID-19: The Relationship of Organizational Support, Organizational Commitment, and Turnover Intention. *International Journal of Environmental Research and Public Health*.

[39] Page K., Graves N. (2021). A Cross Sectional Study of Organizational Factors and Their Impact on Job Satisfaction and Emotional Burnout in a Group of Australian Nurses: Infection Control Practitioners. *BMC Health Services Research*.

[40] Kelly L. A., Gee P. M., Butler R. J. (2021). Impact of Nurse Burnout on Organizational and Position Turnover. *Nursing Outlook*.

[41] Wolf E. J., Harrington K. M., Clark S. L., Miller M. W. (2013). Sample Size Requirements for Structural Equation Models: An Evaluation of Power, Bias, and Solution Propriety. *Educational and Psychological Measurement*.

[42] Su Z., Li Y., Xie Y. (2024). Acute and Long COVID-19 Symptoms and Associated Factors in the Omicron-Dominant Period: A Nationwide Survey via the Online Platform Wenjuanxing in China. *BMC Public Health*.

[43] von Elm E., Altman D. G., Egger M. (2008). The Strengthening the Reporting of Observational Studies in Epidemiology (STROBE) Statement: Guidelines for Reporting Observational Studies. *Journal of Clinical Epidemiology*.

[44] Li J., Li D. (2003). Research on the Relationship between Role Conflict, Organizational Commitment and Turnover Intention under the Rectangular Organizational Structure. Take Industrial Technology Research Staff as an Example. *Chinese Nursing Education*.

[45] Mao G., Liu W., Song H. (2014). Research on the Perception of Decent Work: Scale Development and Testing. *Statistics and Decision*.

[46] Gao J., Liu Y., Xie W., Wang Y., Liao R., Diao H. (2014). The Reliability and Validity of the Chinese Version of the Colleague Solidarity of Nurses’ Scale. *Chinese Journal of Nursing*.

[47] Schumacker R. E., Lomax R. G. (1996). *A Beginner’s Guide to Structural Equation Modeling*.

[48] Byrne B. M. (2016). *Structural Equation Modeling With AMOS: Basic Concepts, Applications, and Programming*.

[49] Lawal L., Lawal A. O., Amosu O. P. (2022). The COVID-19 Pandemic and Health Workforce Brain Drain in Nigeria. *International Journal for Equity in Health*.

[50] Xie Z. (1994). Regarding Men as Superior to Women: Impacts of Confucianism on Family Norms in China. *China Population Today*.

[51] Zhang H., Tu J. (2020). The Working Experiences of Male Nurses in China: Implications for Male Nurse Recruitment and Retention. *Journal of Nursing Management*.

[52] Stanley D., Beament T., Falconer D. (2016). The Male of the Species: A Profile of Men in Nursing. *Journal of Advanced Nursing*.

[53] Shen J., Guo Y., Chen X., Tong L., Lei G., Zhang X. (2022). Male Nurses’ Work Performance: A Cross Sectional Study. *Medicine*.

[54] Liu T., Xu M., Ye M. (2022). Meanings and Senses of Organisational Silence by Male Nurses in the Emergency Department: An Interpretative Phenomenological Study Protocol. *BMJ Open*.

[55] American Public Health Association (2023). Support Decent Work for All as a Public Health Goal in the United States, (APHA Policy Statement Number 20223, Adopted November 2022). *New Solutions: A Journal of Environmental and Occupational Health Policy: NS*.

[56] Zheng J., Feng S., Gao R. (2024). The Relationship Between Organizational Support, Professional Quality of Life, Decent Work, and Professional Well-Being Among Nurses: A Cross-Sectional Study. *BMC Nursing*.

[57] Leineweber C., Bernhard-Oettel C., Eib C., Peristera P., Li J. (2021). The Mediating Effect of Exhaustion in the Relationship between Effort-Reward Imbalance and Turnover Intentions: A 4-Year Longitudinal Study from Sweden. *Journal of Occupational Health*.

[58] Hämmig O. (2018). Explaining Burnout and the Intention to Leave the Profession Among Health Professionals: A Cross-Sectional Study in a Hospital Setting in Switzerland. *BMC Health Services Research*.

[59] Chen S., Yu H., Hsu H., Lin F., Lou J. (2013). Organisational Support, Organisational Identification and Organisational Citizenship Behaviour Among Male Nurses. *Journal of Nursing Management*.

[60] Wu C., Cheng S., Wu J. (2023). Factors Influencing Work Engagement Among Male Nurses: A Structural Equation Model. *Nursing Open*.

[61] Pearson M. M. (2020). Transformational Leadership Principles and Tactics for the Nurse Executive to Shift Nursing Culture. *JONA: The Journal of Nursing Administration*.

[62] Yang C., Gau Ml., Shiau Sj., Hu Wh., Shih Fj. (2004). Professional Career Development for Male Nurses. *Journal of Advanced Nursing*.

[63] Pishgooie A. H., Atashzadeh‐Shoorideh F., Falcó‐Pegueroles A., Lotfi Z. (2019). Correlation Between Nursing Managers’ Leadership Styles and Nurses’ Job Stress and Anticipated Turnover. *Journal of Nursing Management*.

[64] Gauci P., Luck L., O’Reilly K., Peters K. (2023). Workplace Gender Discrimination in the Nursing Workforce—An Integrative Review. *Journal of Clinical Nursing*.

[65] Hsu H., Chen S., Yu H., Lou J. (2010). Job Stress, Achievement Motivation and Occupational Burnout Among Male Nurses. *Journal of Advanced Nursing*.

[66] Baker M. J., Fisher M. J., Pryor J. (2023). Male Nurse Practice in Inpatient Rehabilitation. Finding a Safe Way: A Grounded Theory. *International Journal of Nursing Studies*.

