# Supplementary material for: Turnover Intention Among Male Nurses in Jiangsu, China: A Structural Equation Modeling Study Based on Social Cognitive Theory
Source: J Nurs Manag. 2025 May 5;2025:8865799. doi: 10.1155/jonm/8865799 (PMC12069842; doi:10.1155/jonm/8865799)
Supplement: Supporting Information 1 — Informed consent and the translated questionnaire. [file 8865799.f1.docx]

**Informed consent**

A study on the current status of male nurses’ turnover intention

The Honorable Sir:

We are considering inviting you to participate in a study called “A Survey of Male Nurses’ Turnover Intention”. It is important that you understand the details of the study before agreeing to participate. Please read this document carefully and feel free to ask questions. This study has been approved by the healthcare institutional ethics committee and your participation in this study is entirely up to you.

**1. Purpose of the study**

The purpose of this project is to investigate the current situation of male nurses’ turnover intention on their jobs in Jiangsu Province based on social cognitive theory. We aim to analyze the psychological cognitive factors and environmental factors that affect male nurses’ turnover intention on their jobs, in order to formulate a coping strategy accordingly that can effectively reduce male nurses’ turnover intention.

**2. Background**

Across the globe, the presence of male nurses has become increasingly prominent, with their representation among registered nurses reaching 40% in Saudi Arabia, 21% in Italy, 11.1% in Australia, 10.7% in the United Kingdom, and 9.1% in the United States. In China, the proportion of male nurses has also seen an increase, rising to 3% in 2023. Despite this growth, the nursing profession remains predominantly female. The high turnover rate among male nurses, driven by factors such as uncertain career prospects, diminished work value, high workloads, and low social recognition, continues to be a significant challenge. Research indicates that the turnover rates of male nurse are approximately 9.2% in the United States, 37% in Jordan, between 10% and 64.5% in South Korea, and a staggering 38.4% in China. This high turnover rate not only escalates the economic burden of hospital management, as losing a male nurse costs between 0.31 to 1.3 times his annual salary, but also compromises nursing quality and patient safety. Therefore, it is important to understand the current situation of male nurses’ turnover intention, as well as the mechanism of their turnover, and provide practical solutions to minimize the loss of nursing talent, reduce healthcare costs, and promote the development of modern nursing.

At the same time, due to the fragmented analysis of the causes of male nurses’ turnover intention and the lack of scientific theories, it is difficult for existing studies to comprehensively explain the occurrence mechanism of male nurses’ turnover intention. Therefore, based on the social cognitive theory, this study selects colleague solidarity as the environmental influencing factor, and the level of decent work perception as the individual cognitive factors, to comprehensively explore the mechanisms of the behavioral reaction of male nurses’ turnover intention.

**3. Approximate number of subjects and expected duration of participation in the study**

This study will be a cross-sectional survey of all male nurses in Jiangsu Province, and you will participate in this study by answering a two-part questionnaire in your mailbox. It will take approximately 8-10 minutes of your time to fill out the two parts.

**4. The research process**

With the approval of nursing managers, emails were sent to registered male nurses who met the study’s inclusion and exclusion criteria, inviting them to anonymously participate in the study. The questionnaire consists of two main parts, the first part is the participant’s informed consent and an introduction to the purpose and significance of the study; and the second part is socio-demographic information questionnaire, Turnover Intention Scale, Decent Work Perception Scale, and Colleague Solidarity Scale for Nurses. The questionnaires were sent twice, the first time on the day of the male nurses’ participation and the second time as a reminder 10 days later. Afterwards, we will carry out the analysis and discussion of the data in 2024.

**5. Possible risks and discomforts of participating in research**

There are no additional interventions in this study, so there will be no risks to you by participating in this study. To minimize risks, your personal information will be kept confidential in accordance with the relevant regulations.

**6. Potential benefits**

Your participation in this study may not benefit you. However, you can learn more about why male nurses’ desire to leave occurs through the information gained from the study, and the strategies constructed in this study have the potential to provide avenues for future enhancement of the professional environment as well as career development for male nurses.

**7. Costs and compensation**

The study does not include any fees, and the subjects will not receive any payment.

**8. Confidentiality and privacy authorization**

The investigator is responsible for processing your research data in compliance with applicable data protection regulations. However, the Ethics Committee and the higher administration will have access to the data. The results of the study may be published in medical journals/conferences, but your identity will not be disclosed.

By signing this informed consent form, you are giving consent to the researcher to collect, use and share your health information data. Your authorization to allow us to use your health information remains in effect until the study is completed and the results of the study are available. **However, you may withdraw informed consent at any time through the study researchers.**

**9. Voluntary participation/full or partial withdrawal from the study**

Participation in this study is entirely of your own volition. You may choose not to participate in this study, and you are free to withdraw at any time, without prejudice to any of your rights and interests and without discrimination of any kind.

**10. Questions and information**

All members of the research team will answer all your questions before you sign this consent form. If you still have questions, suggestions, or comments after you have signed this consent form, you will also be able to talk to the researchers. You will be able to keep up with information about this study and the progress of the study.

**11. Informed consent**

I was given full opportunity to discuss and ask questions about the above study. I agree to participate in this study and understand that my participation in the study is entirely voluntary. I understand that I may withdraw from the study at any time and that my withdrawal will not affect my future treatment. By signing this informed consent form, I agree that my personal information data, including my medical information data, will be used as described above.

**Questionnaire on the Occupational Status of Male Nurses in Jiangsu Province**

Dear Fellow Male Nurses:

Hello!

This questionnaire is an anonymous survey of registered male nurses in Jiangsu Province. Please read each question and requirement carefully and make the most appropriate choice according to the actual situation. There is no right or wrong answer to the questions. Thank you for your support and cooperation! We wish you a smooth work and happy life!

**I. Basic information**

Below is the section on basic personal information, please check the appropriate box.

1. Age: years (please give the rounded number)

2. Education level: 🞎 Doctor 🞎 Master 🞎 Bachelor 🞎 College Diploma 🞎 Technical secondary school and below

3. Marital status: 🞎 Unmarried 🞎 Married 🞎 Divorced or other

4. Are you an only child? 🞎 Yes 🞎 No

5. How many children do you have? 🞎 None 🞎 One 🞎 Two or more

6. How many years have you worked as a nurse? (rounded)

7. Title: 🞎 Nurse 🞎 Senior nurse 🞎 Supervisor nurse 🞎 Associate chief nurse 🞎 Chief nurse

8. Employment form: 🞎 Permanent employee 🞎 Contract worker 🞎 Dispatched labor

9. What is the nature of your hospital? 🞎 Tertiary hospital 🞎 Secondary hospital 🞎 Primary hospital 🞎Specialized hospital

10. Which department do you work in? 🞎 Internal Medicine 🞎 Surgery 🞎 Obstetrics and Gynecology 🞎 Emergency 🞎 Outpatient 🞎 ICU 🞎 Operating Room 🞎 Anesthesiology 🞎 Pediatrics 🞎 Hemodialysis 🞎 Others:

11. What is your annual income (in RMB, choose the best estimate)? 🞎 ≤50,000 🞎 60,000-100,000 🞎 110,000-150,000 🞎 160,000-200,000 🞎 210,000-250,000 🞎 >250,000

12. Are you a clinical teacher? 🞎 Yes🞎 No

13. Are you a clinical nurse specialist? 🞎 No 🞎 Municipal 🞎 Provincial 🞎 National 🞎 Other:

14. How many night shifts do you work each month? 🞎 None 🞎 1-4 🞎 5-9 🞎 ≥10

**II. Decent work perception**

Please choose the description that matches your situation.

| **Question** | **(1)** | **(2)** | **(3)** | **(4)** | **(5)** |  |
| --- | --- | --- | --- | --- | --- | --- |
| 1. Compared to my basic needs in life, my income from work is | seriously insufficient | not enough | just fine | more than enough | plentiful |  |
| 2. Compared to the local average, the income from my job is | very low | relatively low | average | relatively high | very high |  |
| 3. In recent years, the income from my work | dropped markedly | dropped slightly | did not change | grew but did not meet my expectation | grew and met my expectation |  |
| 4. The benefits provided to me by my organization are | very few | relatively few | average | relatively generous | very generous |  |
| Please rate the following according to your situation. | | | | | | |
|  | **I totally disagree.** | **I somewhat disagree.** | **It’s hard to say.** | **I largely agree.** | **I completely agree.** |  |
| 5. My workload is so heavy that I am constantly overloaded |  |  |  |  |  |  |
| 6. I need to work overtime frequently |  |  |  |  |  |  |
| 7. My workplace environment is crowded, noisy, or boring |  |  |  |  |  |  |
| 8. My career or job advancement in recent years has not met my expectations |  |  |  |  |  |  |
| 9. I feel that the promotion path in my organization is clear |  |  |  |  |  |  |
| 10. I received job-related training at my organization |  |  |  |  |  |  |
| 11. My current employer and/or occupation make me look good in front of my family and friends |  |  |  |  |  |  |
| 12. My current employer and/or occupation make me look good in front of my peers |  |  |  |  |  |  |
| 13. My relatives, friends, and colleagues are envious of my current job |  |  |  |  |  |  |
| 14. My coworkers respect me at work |  |  |  |  |  |  |
| 15. I feel that my boss/supervisor cares about my personal circumstances |  |  |  |  |  |  |
| 16. The working atmosphere is positive |  |  |  |  |  |  |

**III. Turnover intention scale**

| **Question** | **Never**  **(1)** | **Rarely**  **(2)** | **Sometimes**  **(3)** | **Often**  **(4)** |
| --- | --- | --- | --- | --- |
| 1. Are you considering quitting your current job? |  |  |  |  |
| 2. Are you looking for other jobs of the same nature? |  |  |  |  |
| 3. Are you looking for other jobs of a different nature? |  |  |  |  |
| 4. Given your current situation and qualifications, what do you think is the likelihood of finding a suitable position in another organization? |  |  |  |  |
| 5. If you know of a job opening at another organization that would be a good fit for you, how likely do you think you are to get the job? |  |  |  |  |
| 6. Will you quit your current job? |  |  |  |  |

**IV. Colleague solidarity for nurses**

| **Question** | **I totally disagree. (5)** | **I somewhat disagree. (4)** | **I’m not sure. (3)** | **I largely agree. (2)** | **I completely agree. (1)** |
| --- | --- | --- | --- | --- | --- |
| **Head nurse support** |  |  |  |  |  |
| 1. The nurse manager took time to understand my goals and expectations |  |  |  |  |  |
| 2. The nurse manager cares if I am achieving my goals |  |  |  |  |  |
| 3. The nurse manager is looking out for various opportunities in the hospital that can benefit my career development |  |  |  |  |  |
| 4. The nurse manager makes sure that I get complimented after I complete important tasks |  |  |  |  |  |
| 5. The nurse manager provides me with effective feedback on my work |  |  |  |  |  |
| 6. The nurse manager gives me advice on how to improve my work when I am in need |  |  |  |  |  |
| 7. The head nurse supports my desire to obtain additional training or education for future development |  |  |  |  |  |
| 8. The head nurse gives me work assignments that provide opportunities to develop new skills |  |  |  |  |  |
| 9. The head nurse gives me special assignments that promote my development in the hospital |  |  |  |  |  |
| **Colleague support** |  |  |  |  |  |
| 10. The help from peer supporters effectively relieves my stress at work |  |  |  |  |  |
| 11. I value the time spent on building a peer support system |  |  |  |  |  |
| 12. My work stress is relieved significantly because a peer support system is created |  |  |  |  |  |
| 13. In departmental group activities, my peer supporters respect and encourage me and do not force me to participate |  |  |  |  |  |
| 14. My peer supporters provide a supportive environment where I can talk about my feelings with confidence |  |  |  |  |  |
| 15. My peer supporters give me all kinds of support, especially the kind that I need |  |  |  |  |  |
| 16. My peer supporters always seem to have difficulty understanding my point of view |  |  |  |  |  |
| 17. My peer supporters always acknowledge and show that they understand my feelings |  |  |  |  |  |
| 18. My peer supporters give me enough time to express my feelings before giving advice |  |  |  |  |  |
| 19. After communicating with my peer supporters, I get a clearer understanding of the problems I am facing |  |  |  |  |  |
| 20. The questions from my peer supporters can help me think better about my problems |  |  |  |  |  |
| 21. My peer supporters use detailed examples to provide feedback and suggestions |  |  |  |  |  |
| 22. After I communicate with my peer supporters, the goals I set are always a little difficult but achievable |  |  |  |  |  |
| 23. After I communicate with my peer supporters, it is important for me to have clear goals |  |  |  |  |  |
| 24. After I communicate with my peer supporters, all specified goals are measurable |  |  |  |  |  |
| 25. My peer supporters can help me develop clear, simple, and feasible action plans |  |  |  |  |  |
| 26. My peer supporters help me focus on how to achieve success instead of studying old problems |  |  |  |  |  |
| 27. My peer supporters always take my action plans seriously |  |  |  |  |  |
| 28. My peer supporters always ask me about the progress toward my goals |  |  |  |  |  |
| 29. My peer supporters always help me tackle the drawbacks in my work |  |  |  |  |  |
| 30. My peer supporters always affirm my progress and success and encourage me |  |  |  |  |  |
