# Supplementary material for: Turnover Intention Among Male Nurses in Jiangsu, China: A Structural Equation Modeling Study Based on Social Cognitive Theory
Source: J Nurs Manag. 2025 May 5;2025:8865799. doi: 10.1155/jonm/8865799 (PMC12069842; doi:10.1155/jonm/8865799)
Supplement: Supporting Information 2 — Figure 1. Initial structural equation model for the turnover intention of male nurses. Table 1. STROBE Statement: Checklist of items that should be included in reports of cross-sectional studies. [file 8865799.f2.docx]

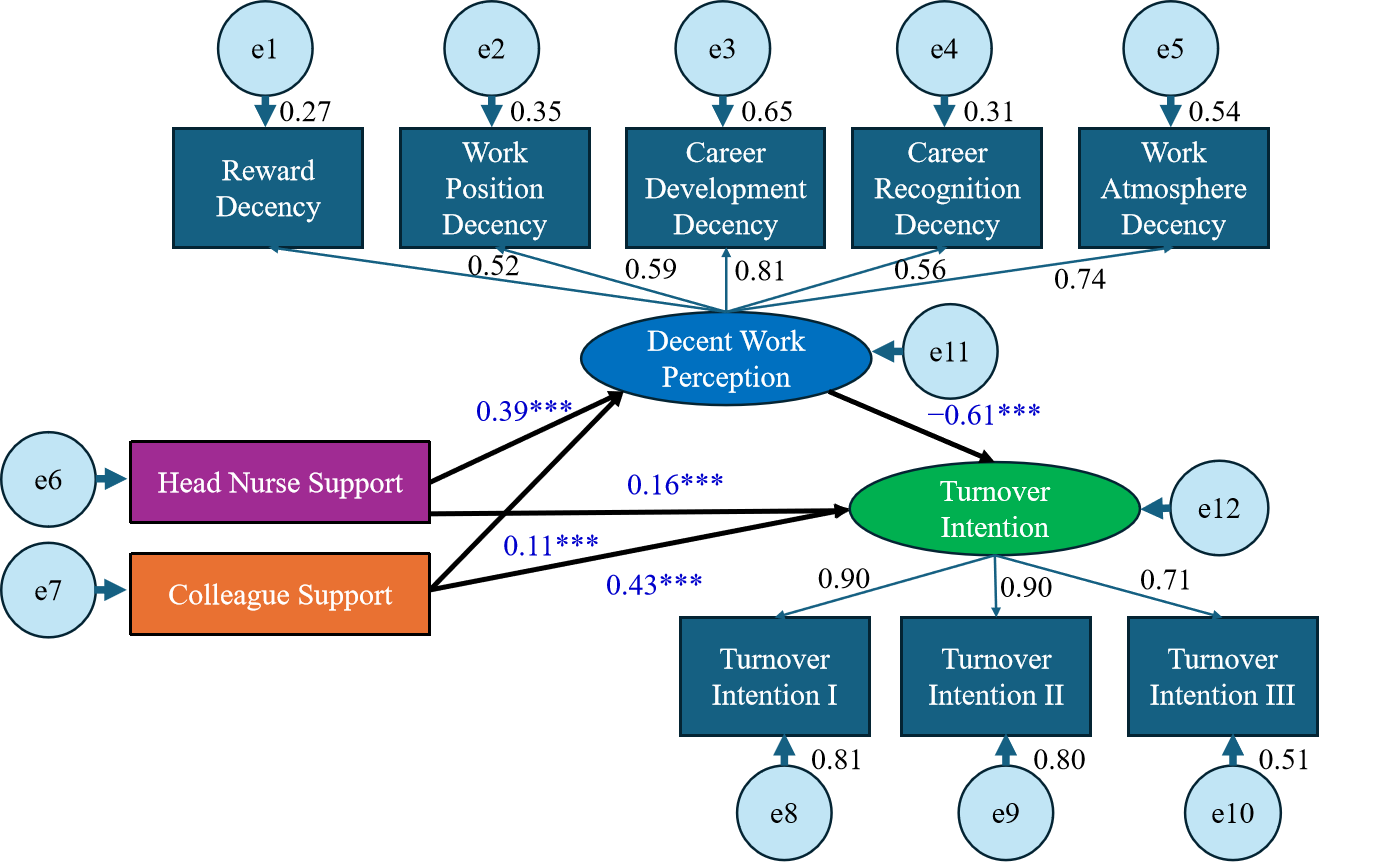


**Figure 1.** Initial structural equation model for the turnover intention of male nurses.

**Table 1** Checklist of items that should be included in reports of cross-sectional studies

|  | | Item No. | Recommendation | Page  No. |
| --- | --- | --- | --- | --- |
| **Title and abstract** | | 1 | (*a*) Indicate the study’s design with a commonly used term in the title or the abstract | Page 2 |
|  |  |  | (*b*) Provide in the abstract an informative and balanced summary of what was done and what was found | Page 2 |
| Introduction | | | | |
| Background/rationale | | 2 | Explain the scientific background and rationale for the investigation being reported | Page 4 |
| Objectives | | 3 | State specific objectives, including any prespecified hypotheses | Line 6-9 |
| Methods | | | | |
| Study design | | 4 | Present key elements of study design early in the paper | Page 10 |
| Setting | | 5 | Describe the setting, locations, and relevant dates, including periods of recruitment, exposure, follow-up, and data collection | Page 10 |
| Participants | | 6 | (*a*) Give the eligibility criteria, and the sources and methods of selection of participants | Page 10 |
| Variables | | 7 | Clearly define all outcomes, exposures, predictors, potential confounders, and effect modifiers. | Page 11-12 |
| Data sources/ measurement | | 8 | For each variable of interest, give sources of data and details of methods of assessment (measurement). Describe comparability of assessment methods if there is more than one group | Page 11-12 |
| Study size | | 9 | Explain how the study size was arrived at | Page 10 |
| Quantitative variables | | 10 | Explain how quantitative variables were handled in the analyses. If applicable, describe which groupings were chosen and why | Page 12-13 |
| Statistical methods | | 11 | (*a*) Describe all statistical methods, including those used to control for confounding | Page 12-13 |
|  |  |  | (*b*) Describe any methods used to examine subgroups and interactions | Page 12 |
|  |  |  | (*c*) Explain how missing data were addressed | Page 11 |
|  |  |  | (*d*) Describe analytical methods taking account of sampling strategy | Page 12 |
| Results | | | | |
| Participants | | 12 | Report numbers of individuals at each stage of study—eg numbers potentially eligible, examined for eligibility, confirmed eligible, included in the study, completing follow-up, and analysed | Page 13 |
| Descriptive data | | 13 | Give characteristics of study participants (eg demographic, clinical, social) and information on exposures and potential confounders | Page 13-15 |
| Outcome data | | 14 | Report numbers of outcome events or summary measures | Page 16 |
| Main results | | 15 | (*a*) Give unadjusted estimates and, confounder-adjusted estimates and their precision (eg, 95% confidence interval). Make clear which confounders were adjusted for and why they were included | Page 18-21 |
|  |  |  | (*b*) Report category boundaries when continuous variables were categorized | Page 20-21 |
| Discussion | | | | |
| Key results | 16 | | Summarise key results with reference to study objectives | Page 21-22 |
| Limitations | 17 | | Discuss limitations of the study, taking into account sources of potential bias or imprecision. Discuss both direction and magnitude of any potential bias | Page 25 |
| Interpretation | 18 | | Give a cautious overall interpretation of results considering objectives, limitations, multiplicity of analyses, results from similar studies, and other relevant evidence | Page 21-24 |
| Generalisability | 19 | | Discuss the generalisability (external validity) of the study results | Page 25 |
| Other information | | |  | |
| Funding | 20 | | Give the source of funding and the role of the funders for the present study and, if applicable, for the original study on which the present article is based | Page 26 |
